# Maspin and MCM2 immunoprofiling in salivary gland carcinomas

**DOI:** 10.1186/1746-1596-6-89

**Published:** 2011-09-26

**Authors:** Shaimaa E Ghazy, Iman M Helmy, Houry M Baghdadi

**Affiliations:** 1 Oral Pathology Department, Faculty of Dentistry, Ain Shams University, Cairo, Egypt

**Keywords:** Maspin, MCM2, salivary gland carcinomas

## Abstract

**Background:**

The pathogenesis of salivary gland carcinomas is very complex and prognostic markers are difficult to find in these carcinomas of which the different subtypes have varying malignant potential. The study was conducted to examine the cellular distribution of maspin and MCM2 in salivary gland carcinomas and their value to predict lymph node metastasis.

**Materials and methods:**

Fifty three paraffin blocks of different lesions (15 muco-epidermoid carcinoma, 14 adenoid cystic carcinoma, 3 epi-myoepithelial carcinoma, 5 salivary duct carcinoma, 5 malignant pleomorphic adenoma, 6 polymorphous low grade adenocarcinoma and 5 acinic cell carcinoma) were prepared for immunohistochemical staining with maspin and MCM2 antibodies. ANOVA and Pearson correlation tests were used for the statistical analysis of the results.

**Results:**

All salivary gland carcinomas express maspin and MCM2 with variable cellular localization. There was a significant difference in the expression of each antibody between mucoepidermoid carcinoma, adenoid cystic carcinoma and polymorphous low grade adenocarcinoma. No association was found between examined markers and lymph node metastasis.

**Conclusions:**

Salivary gland carcinomas express maspin and MCM2 with variable levels and cellular localization, consisting important markers of biological behavior in these tumors. The level of MCM2 expression can be used in the differential diagnosis of adenoid cystic carcinoma and polymorphous low grade adenocarcinoma. Further study with large sample size is recommended to assess their value in prediction of lymph node metastasis.

## Introduction

Salivary gland neoplasms which comprise about 5% of head and neck cancers are a morphologically and clinically diverse group of lesions and may present considerable diagnostic challenge to the pathologist [1]. The most frequent salivary gland carcinoma types are mucoepidermoid carcinoma, adenoid cystic carcinoma, acinic cell carcinoma, malignant pleomorphic adenoma and salivary duct carcinoma [2].

Mammary Serine Protease Inhibitor (maspin) belongs to the serine protease inhibitor (serpin) family [3,4], which comprises a large protein family with diverse biological functions [5]. There is a controversy about maspin protease inhibition; Sheng et al [6] stated that maspin has protease inhibitory activity. On the contrary, Bass et al [7] reported that maspin has no protease inhibitory properties.

Maspin expression has been demonstrated in multiple tissues including epithelium of the breast, prostate, lung and in stromal cells of the cornea [8-10]. Maspin demonstrates broad localization patterns [5], in mammary epithelial cells, maspin localizes primarily to the cytoplasm, but can also localize to the nucleus, and the cell surface [11].

One of the first regulatory mechanisms identified for maspin involved p53 signaling. The regulation of maspin by p53 could explain the role of p53 in cell invasion and metastasis and hypothesizes that cancer cells expressing mutant p53 would be more likely to metastasize, in part due to the inability to upregulate the maspin gene [5]. In addition, increased maspin was associated with an increase in apoptosis and a reduction in cell invasion. This effect was blocked by the addition of a maspin-blocking antibody [12].

Recent study has also established a role for the common breast cancer drug Tamoxifen (TAM) in regulating the expression of maspin [5]. The clinical efficacy of TAM has been attributed to growth arrest and induction of apoptosis in breast cancer cells. TAM was shown to induce maspin expression in vitro and in situ [13]. Also, it was suggested that maspin has an inhibitory effect on tumor induced angiogenesis [14], cell motility, invasion and metastasis [15]. Extensive studies have been undertaken to determine the mechanisms employed by maspin to produce its anti-metastatic effects. One line of evidence suggests that maspin regulates cell invasion by altering the integrin profile of the cell [5]. In support of a cell surface event, it has been reported that cell surface-associated maspin is primarily responsible for its anti-invasive properties [16].

Several reports indicated that maspin can function as an inhibitor of angiogenesis. Both rMaspin and secreted maspin can impede the migration of cultured endothelial cells toward bFGF and VEGF which act as important chemo-attractants during angiogenesis. Also, maspin was shown to effectively block neovascularization and reduce the density of the neoplasm-associated microvessels in vivo [17,18]. Solomon et al [19] reported that neoplasms with both cytoplasmic and nuclear maspin expression had lower VEGF and cyclooxygenase-2 (COX-2) expression than neoplasms with cytoplasmic maspin expression only, so suppression of VEGF by maspin may thus occur through a COX-2 mediated pathway.

In addition to its anti-angiogenic properties, maspin has also been implicated in apoptosis [5]. It has been demonstrated that maspin sensitizes breast cancer cells to staurosporine (STS)-induced apoptosis [20]. Staurosporine is a synthetic chemical known to induce apoptosis via an intrinsic pathway [21]. The apoptotic effect of maspin appears to be tumor-specific since normal epithelial cells that express maspin at a high level are not sensitized to drug-induced apoptosis [22].

The ubiquitous localization of maspin (cytoplasmic, nuclear, cell surface-associated, secreted) suggests that maspin may be involved in multiple pathways and processes. Loss of maspin has been associated with poor prognosis in various malignant neoplasms like ovarian cancer, oral squamous cell carcinoma, lung and prostate cancer [23-25].

The MCM (minichromosome maintance) proteins identify a group of ten conserved factors functioning in the replication of the genome of eukaryotic organisms [26]. Among these, MCM2-7 proteins are related to each other and form a complex implicated at the initiation step of DNA synthesis. MCM2-7 act as licensing factors for DNA replication to ensure that the genome is replicated only once in each cell cycle [26,27].

Since MCM activity is essential for DNA replication in dividing cells and is lost in quiescence [28], MCMs are obvious markers for proliferation [26]. Molecular studies suggested that increased levels of MCMs mark not only proliferative malignant cells, but also precancerous cells and the potential for recurrence [29,30]. In breast cancers, increasing neoplasm grade is associated with increased MCM2 expression [31]. Thus, they may prove to be effective markers for diagnosis of neoplasms [26].

Several studies reported that MCM2 and Ki-67 are both markers of cellular proliferation and required for cell cycle progression [32]. They showed that anti-MCM2 antibody stained a larger number of cells than anti Ki-67, suggesting that Ki-67 may be expressed during a shorter interval of the cell cycle than MCM2 [33]. MCM2 is present throughout the four phases of cell cycle [31], while Ki-67 is predominantly expressed during S, G2 and M phases. The present study aimed to evaluate the expression of maspin and MCM2 in salivary gland carcinomas and their value to predict lymph node metastasis.

## Materials and methods

The material of this study consisted of 53 formalin-fixed, paraffin-embedded specimens of malignant salivary gland neoplasms, all collected from the archives of the General Pathology Department, Faculty of Medicine, Ain Shams University and National Cancer Institute, Cairo University. Fifteen cases of mucoepidermoid carcinoma (MEC), 10 of which were diagnosed as high grade and the other 5 cases as low grade, 14 cases of adenoid cystic carcinoma (ADCC), 5 cases of salivary duct carcinoma (SDC), 3 cases of epi-myoepithelial carcinoma (EMC), 5 cases of malignant pleomorphic adenoma (MPA), 6 cases of polymorphous low-grade adenocarcinoma (PLGA) and 5 cases of acinic cell carcinoma (ACC). Clinical information about lymph node metastasis was obtained from patients' medical records (summary of cases is displayed in table 1). For all specimens five micrometer thick sections were prepared and stained with hematoxylin and eosin to confirm the diagnosis.

**Table 1:** Types of cases and association with lymph node metastasis of the selected cases.

Malignant Salivary Gland Neoplasms	Total Number of cases	Number of cases Associated with lymph node metastasis
MEC	15	4 (high grade variant)

ADCC	14	4 (2 of solid type and 2 of cribriform type)

EMC	3	-

SDC	5	2

M PA	5	-

PLGA	6	-

ACC	5	1

Total	53	11

In summary, immunohistochemical staining is performed as follows: the tissues were deparaffinized in xylene and hydrated through graded alcohol and washed with tap water. Based on the manufacturers' recommendation, the slides were transferred for antigen retrieval. The slides were washed with phosphate buffer and separately incubated with maspin (Visionbiosystems Novocastra™ Laboratories, Ltd, United Kingdom) diluted at a ratio of 1:30 and MCM2 (Lab Vision Corporation, USA) diluted at a ratio of 1:50 over-night. The slides were then washed again in PBS and incubated with biotinylated antibody for 30 minutes, and then were washed in PBS. Finally, the slides were incubated with peroxidase labeled streptavidin for 30 minutes and washed in PBS. Subsequently, DAB chromogen was applied for antibody staining (brown). The samples were then allowed to react with Mayer's hematoxylin for 5 minutes, dehydrated and covered with cover glass.

For each positive section, 6 microscopic fields showing highest immunopositivity were selected and photomicrographs were captured at original magnification 40×. This was performed using a digital camera (C5060, Olympus, Japan) mounted by a C-mount to a light microscope (BX60, Olympus, Japan). All the steps for immunohistochemical evaluation were carried out using image analysis software (Image J, 1.41a, NIH, USA). The area fraction (AF) of the positive cells was calculated automatically. The area fraction represented the percentage of immunopositive area to the total area of the microscopic field. The collected data was tabulated in an excel sheet and statistically analyzed using SPSS 15.

## Results

**Figure 1:**
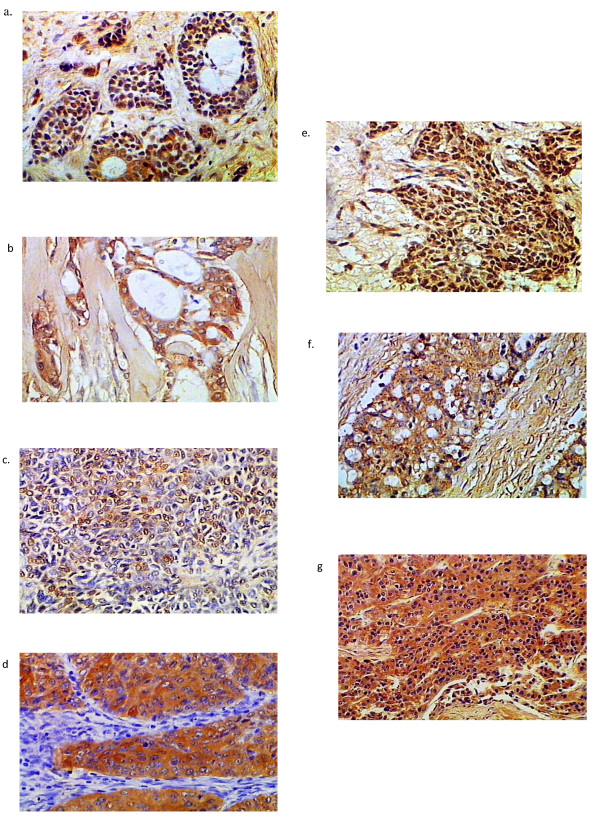
**Maspin expression patterns in salivary gland carcinomas**. a: low grade MECx200. b: ADCCx200. c: EMCx200. d: SDCx200. e: MPAx200. f: PLGAx200. g: ACCx100.

**Figure 2:**
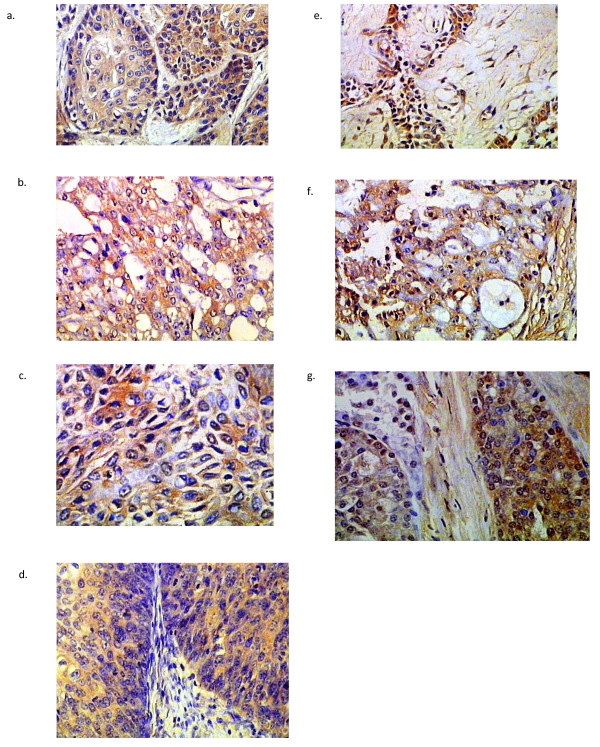
**MCM2 expression patterns in salivary gland carcinomas**. a: high grade MECx200. b: ADCCx200. c: EMCx400. d: SDCx200. e: MPAx200. f: PLGAx200. g: ACCx400.

All cases of MEC demonstrated maspin immunoreactivity. Most of the epidermoid cells in the cases diagnosed as high grade were negative for maspin, with a few positive cells in which the reaction was cytoplasmic. The mucous-secreting cells showed membranous staining. Low grade MEC showed more positive cells with nuclear and cytoplasmic staining of the epidermoid cells (Figure 1a). Concerning MCM2, most of the epidermoid cells revealed cytoplasmic staining in high grade cases (Figure 2a), while in low grade cases, little number of epidermoid cells were positive.

In ADCC, 12 cases of cribriform pattern demonstrated cytoplasmic maspin and MCM2 staining (Figure 1b, 2b). Two cases of solid pattern were negative for maspin but revealed cytoplasmic reaction for MCM2. Myoepithelial cells were negative for both antibodies.

In EMC, the neoplastic cells demonstrated cytoplasmic and nuclear (either the whole nucleus or confined only to the nuclear membrane) maspin staining and cytoplasmic MCM2 staining (Figure 1c, 2c). Few neoplastic cells were negative for maspin and MCM2.

In SDC, cases with solid and papillary pattern showed cytoplasmic maspin and MCM2 reaction (Figure 1d, 2d). In cribriform pattern with comedo necrosis, little nuclear and mainly cytoplasmic maspin reaction was detected, while MCM2 revealed cytoplasmic reaction only.

In MPA, all cases revealed nuclear and cytoplasmic maspin and MCM2 reaction in epithelial and some myoepithelial cells (Figure 1e, 2e), but in PLGA, there was cytoplasmic reaction for both antibodies in neoplastic cells arranged in cribriform and cystic pattern (Figure 1f, 2f). In ACC, one case of clear cell variant demonstrated membranous maspin and MCM2 reaction, while the remaining cases showed nuclear and cytoplasmic reaction for both antibodies (Figure 1g, 2g).

Statistically, ANOVA test revealed significant difference in the expression of both markers between MEC, ADCC and PLGA (P value = 0.000) (table 2, 3). Results of Post Hoc test for maspin and MCM2 expression are shown in table 4 and 5 respectively. Pearson's correlation analysis revealed no significant correlation between both maspin or MCM2 expression and lymph node metastasis.

**Table 2:** ANOVA for maspin

ANOVA					
MEAN AF					
	**Sum of Squares**	**df**	**Mean Square**	**F**	**Sig**.

Between Groups	3682.051	2	1841.026	683.744	.000
Within Groups	80.777	30	2.693		
Total	3762.828	32			

**Table 3:** ANOVA for MCM2

ANOVA					
MEAN AF					
	**Sum of Squares**	**df**	**Mean Square**	**F**	**Sig**.

Between Groups	713.006	2	356.503	39.753	.000
Within Groups	286.978	32	8.968		
Total	999.984	34			

**Table 4:** Post Hoc Test for maspin

Multiple Comparisons						
MEAN AF Tukey HSD						
**(I) type of lesion**	**(J) type of lesion**	**Mean Difference (I-J)**	**Std. Error**	**Sig**.	**95% Confidence Interval**	
					**Lower Bound**	**Upper Bound**

MEC	adcc	-9.40967*	.63552	.000	-10.9764	-7.8429
	Poly LG AdenoCa	-29.27800*	.79263	.000	-31.2321	-27.3239

adcc	MEC	9.40967*	.63552	.000	7.8429	10.9764
	Poly LG AdenoCa	-19.86833*	.82045	.000	-21.8910	-17.8457

Poly LG AdenoCa	MEC	29.27800*	.79263	.000	27.3239	31.2321
	adcc	19.86833*	.82045	.000	17.8457	21.8910

*. The mean difference is significant at the 0.05 level.

**Table 5:** Post Hoc Test for MCM2

Multiple Comparisons						
MEAN AF Tukey HSD						
**(I) type of lesion**	**(J) type of lesion**	**Mean Difference (I-J)**	**Std. Error**	**Sig**.	**95% Confidence Interval**	
					**Lower Bound**	**Upper Bound**

MEC	adcc	-2.52957	1.11285	.074	-5.2643	.2051
	Poly LG AdenoCa	10.35900*	1.44656	.000	6.8043	13.9137

adcc	MEC	2.52957	1.11285	.074	-.2051	5.2643
	Poly LG AdenoCa	12.88857*	1.46125	.000	9.2977	16.4794

Poly LG AdenoCa	MEC	-10.35900*	1.44656	.000	-13.9137	-6.8043
	adcc	-12.88857*	1.46125	.000	-16.4794	-9.2977

*. The mean difference is significant at the 0.05 level.

## Discussion

Salivary gland neoplasms are a relatively rare and morphologically diverse group of lesions. A diagnosis based on hematoxylin and eosin stained sections remains the gold standard in salivary gland pathology [1].

Several studies were performed to detect the expression of maspin protein in different malignant neoplasms such as ovarian carcinoma, oral squamous cell carcinoma, pulmonary adenocarcinoma and prostate carcinoma to clarify its role in malignancy. Most researchers found that the increased maspin expression does correlate with better prognosis in these neoplasms [19,23-25].

Recent studies have proposed that MCM proteins may be sensitive proliferation markers and may serve as novel biomarkers for prognostication and diagnosis of various premalignant and malignant lesions [31,34,35]. The superior sensitivity of the MCM proteins over the standard proliferation markers such as Ki-67 resides in the fact that MCMs identify not only cycling cells, but also non-cycling cells with proliferative potential [35].

High grade MEC is characterized by decreased maspin expression. Loss or decreased expression of maspin indicates loss of its role in inhibition of tumor invasion, metastasis and angiogenesis [3]. Evidence of both nuclear and cytoplasmic maspin expression was observed in low grade MEC; this finding denotes that maspin cellular localization has an influence on its role as tumor suppressor gene [5]. Sood et al [36] demonstrated that mixed nuclear and cytoplasmic maspin localization in ovarian cancer is indicative of a more benign lesion than neoplasms with cytoplasmic expression only, suggesting an important tumor-suppressive role for nuclear maspin. This maspin nuclear localization pattern had been seen in other neoplasms as well, including non-small cell lung carcinoma and pancreatic cancer, where predominantly nuclear maspin is associated with favorable morphologic features [11,24].

The high grade MEC cases showed a marked increase of MCM2 expression when compared to the low grade cases, which was also in agreement with Vargas et al [34]. This might be explained by the fact that in cancer, differentiated neoplastic cells tend to grow and spread at a slower rate than undifferentiated or poorly differentiated cells, which lack the structure and function of normal cells and grow uncontrollably [37]. It was stated that withdrawal of cells from the cell cycle into differentiated state is coupled with downregulation of MCM2 expression [31,35].

In ADCC, cases with solid pattern were maspin immunonegative, which was in agreement with Navarro et al [15] and indicates the aggressive behavior of this pattern. Maass et al [38] stated that maspin is lost in the metastatic cells, and Shwarz et al [3] reported in his study that in intermediate grade tumours (ADCC, MEC, carcinoma expleomorphic adenoma) loss of maspin expression, mainly localized to the nucleus, was associated with lymph node metastases.

Myoepithelial cells in ADCC were maspin immunonegative. This result was in agreement with Navarro et al [15]. Normally maspin is expressed in high amounts in myoepithelial cells and it was suggested that maspin can be used as a myoepithelial cell marker [39]. Thus, loss of maspin in this cell might indicate the role of myoepithelial cells in malignant transformation and histogenesis of this neoplasm [40].

Adenoid cystic carcinoma demonstrated high value of MCM2 expression; this finding gives an impression of the high proliferative power of ADCC. This result was consistent with previous study denoting that ADCC is a highly proliferative salivary gland neoplasm [34].

Myoepithelial carcinoma showed the smallest value of maspin expression, and high value of MCM2 expression. These values might indicate the aggressive behavior of this neoplasm. The knowledge of myoepithelial carcinoma behavior and the optimal line of management are deficient, possibly due to its rare occurrence and the lack of comprehensive reports of large case series [41]. However, some authors describe myoepithelial carcinoma by infiltrative growth and potential metastasis [2,41,42].

Salivary duct carcinoma revealed the highest value of MCM2 expression and reduced maspin expression; this finding confirms the high grade behavior of this tumor [43].

Malignant pleomorphic adenoma possessed the highest value of maspin expression and low MCM2 expression, this is in accordance with Umekita et al [44] who observed that high maspin expression in breast carcinoma was associated with poor prognosis, and this was explained by genetic alteration at the maspin gene locus contributing to the loss of tumor suppressing function of the maspin protein. Values of maspin and MCM2 expression in PLGA and ACC reflected low grade nature of these tumors which are characterized by a low metastatic potential and a high survival rate [2].

The differential diagnosis of both ADCC and PLGA is of a great interest as both share common histopathological features and may cause diagnostic difficulty particularly in small biopsies [1]. Our data demonstrated that ADCC has a higher proliferation power compared to PLGA as determined by MCM2 immunostaining. This result was in conformity with Vargas et al [34] who support the idea that proliferation markers can be used to differentiate borderline cases.

No significant correlation between maspin expression and lymph node metastasis was found in this study. This finding disagrees with Schwarz et al [3] who reported that lack of maspin expression was significantly correlated with positive lymph node metastasis. This might be due to the small sample size used in this study in which only 11 cases were associated with positive lymph node metastasis.

Also, there was no significant correlation between MCM2 expression and positive lymph node metastasis. This result was in agreement with Vargas et al [34] and was dissimilar to Guzinska-Ustymowiczi et al study [45] in which MCM2 over expression was associated with lymph node metastasis.

Maspin and MCM2 are important markers of biological behavior in salivary gland carcinomas. Further study with large sample size is required to clarify the correlation between maspin and MCM2 expression and lymph node metastasis.

## Competing interests

The authors declare that they have no competing interests.

## Authors' contributions

SE participated in collection of data and references, immunohistochemical staining and evaluation by image analysis. IH participated in the design of the study, performed the statistical analysis and drafted the manuscript. HB participated in the design and coordination, sequence alignment and reviewing the manuscript. All authors read and approved the final manuscript.
